# Hypomorphic *GINS3* variants alter DNA replication and cause Meier-Gorlin syndrome

**DOI:** 10.1172/jci.insight.155648

**Published:** 2022-05-23

**Authors:** Mary E. McQuaid, Kashif Ahmed, Stephanie Tran, Justine Rousseau, Ranad Shaheen, Kristin D. Kernohan, Kyoko E. Yuki, Prerna Grover, Ema S. Dreseris, Sameen Ahmed, Lucie Dupuis, Jennifer Stimec, Mary Shago, Zuhair N. Al-Hassnan, Roch Tremblay, Philipp G. Maass, Michael D. Wilson, Eyal Grunebaum, Kym M. Boycott, François-Michel Boisvert, Sateesh Maddirevula, Eissa A. Faqeih, Fahad Almanjomi, Zaheer Ullah Khan, Fowzan S. Alkuraya, Philippe M. Campeau, Peter Kannu, Eric I. Campos, Hugo Wurtele

**Affiliations:** 1 Maisonneuve-Rosemont Hospital Research Center, Montreal, Quebec, Canada.; 2 Peter Gilgan Centre for Research and Learning, The Hospital for Sick Children, Toronto, Ontario, Canada.; 3 Department of Molecular Genetics, University of Toronto, Toronto, Ontario, Canada.; 4 CHU Sainte-Justine, Montreal, Quebec, Canada.; 5 Department of Translational Genomics, Center for Genomic Medicine, King Faisal Specialist Hospital and Research Center, Riyadh, Saudi Arabia.; 6 CHEO Research Institute, Ottawa, Ontario, Canada.; 7 Newborn Screening Ontario, CHEO, Ottawa, Ontario, Canada.; 8 University of Sherbrooke, Sherbrooke, Quebec, Canada.; 9 Section of Medical Genetics, Children’s Specialist Hospital, and; 10 Department of Pediatric Hematology and Oncology, Comprehensive Cancer Center, King Fahad Medical City, Riyadh, Saudi Arabia.; 11 Department of Anatomy and Cell Biology, College of Medicine, Alfaisal University, Riyadh, Saudi Arabia.; 12 Department of Medical Genetics, University of Alberta, Edmonton, Alberta, Canada.; 13 Department of Medicine, University of Montreal, Montreal, Quebec, Canada.

**Keywords:** Cell Biology, Cell cycle, DNA repair, Genetic diseases

## Abstract

The eukaryotic CDC45/MCM2-7/GINS (CMG) helicase unwinds the DNA double helix during DNA replication. The GINS subcomplex is required for helicase activity and is, therefore, essential for DNA replication and cell viability. Here, we report the identification of 7 individuals from 5 unrelated families presenting with a Meier-Gorlin syndrome–like (MGS-like) phenotype associated with hypomorphic variants of *GINS3*, a gene not previously associated with this syndrome. We found that MGS-associated *GINS3* variants affecting aspartic acid 24 (D24) compromised cell proliferation and caused accumulation of cells in S phase. These variants shortened the protein half-life, altered key protein interactions at the replisome, and negatively influenced DNA replication fork progression. Yeast expressing MGS-associated variants of *PSF3* (the yeast *GINS3* ortholog) also displayed impaired growth, S phase progression defects, and decreased Psf3 protein stability. We further showed that mouse embryos homozygous for a D24 variant presented intrauterine growth retardation and did not survive to birth, and that fibroblasts derived from these embryos displayed accelerated cellular senescence. Taken together, our findings implicate *GINS3* in the pathogenesis of MGS and support the notion that hypomorphic variants identified in this gene impaired cell and organismal growth by compromising DNA replication.

## Introduction

In eukaryotes, the 11-subunit holoenzyme known as the CMG (CDC45/MCM2-7/GINS) helicase (1–3) separates template DNA strands to allow DNA replication. In the G1 phase of the cell cycle, 2 MCM2-7 hexameric rings are loaded in a head-to-head configuration at DNA sequences called origins of replication (4); however, the helicase activity of the MCM ATPases is weak in the absence of the other proteins that compose the CMG (5). In S phase, origins of replication are activated when CDC45 and the GINS subcomplex associate with MCM2-7, thereby stimulating its activity (6). Once activated, 2 CMG helicases create a bidirectional replication bubble at origins, with each helicase tracking 3′ to 5′ on the leading strand (7, 8).

The GINS subcomplex is evolutionarily conserved in eukaryotes ranging from yeast to human and is composed of the GINS1/PSF1, GINS2/PSF2, GINS3/PSF3, and GINS4/SLD5 subunits (9). Besides enabling MCM helicase activity, the GINS subcomplex couples the CMG complex to DNA polymerase ε via interactions with GINS1, which stimulates the activity of the former (10–13). Mutations affecting the CMG–polymerase ε interaction (e.g., in the Dbp2 subunit of polymerase ε or in Psf1) have been shown to negatively impact DNA replication fidelity and cell growth in yeast (9, 14, 15). On the lagging strand, the CTF4 (WDHD1/AND-1) trimeric adaptor associates with GINS4, thereby coupling the CMG helicase to DNA polymerase α (16–19).

Genetic variations affecting the CMG helicase or its assembly are associated with Meier-Gorlin syndrome (MGS, also known as “ear, patella, short stature syndrome” and/or “microtia, absent patellae, micrognathia syndrome”), a rare disorder most often inherited in an autosomal recessive manner and characterized by primordial dwarfism, microtia, and small/absent patellae (reviewed in ref. 20). Not all 3 clinical features are required to diagnose MGS, although 97% of MGS patients present at least 2 of these 3 characteristics. Other phenotypes associated with MGS include respiratory and gastrointestinal problems, skeletal and genitourinary anomalies, and facial characteristics such as down-slanting palpebral fissures and full lips (20). Of interest here, pathogenic variants associated with MGS have been identified in the genes encoding subunits of the origin recognition complex ORC1, ORC4, and ORC6 (21); components of the CMG complex MCM3 (22), MCM5 (23), MCM7 (22), CDC45 (24–27), and GINS2 (28); factors involved in the assembly of the CMG complex CDT1, CDC6 (21),and GMNN (29); and DONSON, a protein that promotes replication fork stability (30). Additionally, pathogenic variants in genes encoding CMG components MCM4 (31, 32), MCM7 (22), CDC45 (26), and GINS1 (33, 34) have been associated with other forms of primordial dwarfism.

Pathogenic CMG variants have been shown to influence various facets of DNA replication, cell cycle progression, genomic stability, and organismal development. *MCM4* variants linked to short stature were shown to increase the proportion of cells in G2/M, as well as the frequency of chromosomal aberrations (32). While *MCM4*-null mice are not viable, mice expressing hypomorphic *MCM4* suffered from adrenal insufficiency, although they were of normal size (31). *GINS1* variants linked to short stature and NK cell deficiencies were found to destabilize the CMG helicase complex and to compromise the activation of origins of replication (33). Cells harboring *GINS* variants also show increased replication fork stalling, reduced proliferation rates, elevated DNA damage, accumulation in G2/M, and mitotic defects (33). Finally, a MGS-associated *GINS2* variant was recently shown to cause sensitivity to replicative stress-inducing chemicals in a yeast model (28). While the above data support the notion that compromised CMG function is associated with MGS and short stature in humans, the spectrum of MGS-associated mutations is incompletely characterized. Here, we report *GINS3* variants associated with an MGS-like clinical presentation and characterize their impact on DNA replication, as well as on organismal development, using human, yeast, and murine model systems.

## Results

### GINS3 variants identified in patients with MGS phenotypes.

**Figure 1:**
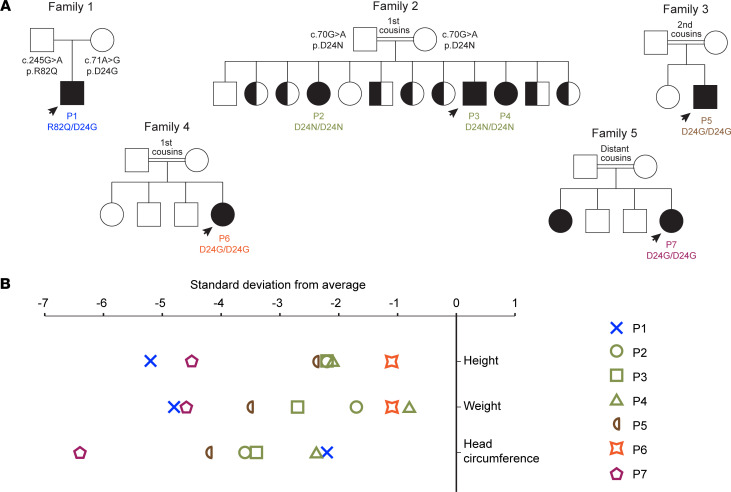
Patients with hypomorphic *GINS3* variants present with growth deficiencies. (**A**) Pedigrees of 5 families showing allele segregation. Probands are indicated by an arrow. (**B**) Growth parameters of 7 individuals with biallelic mutations in *GINS3*.

**Figure 2:**
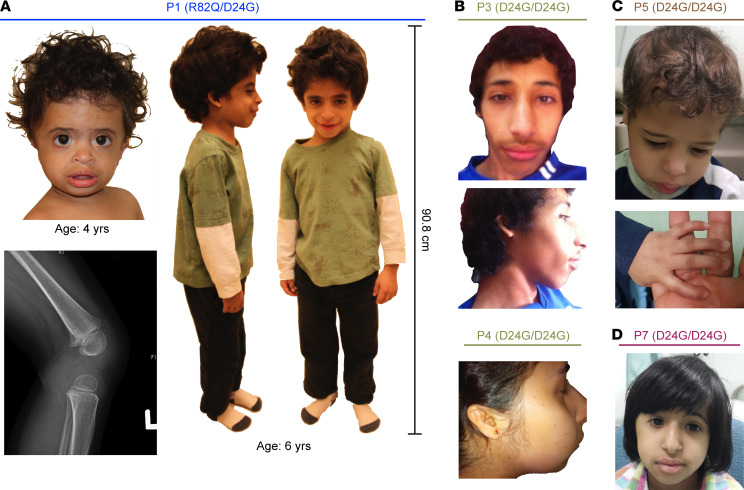
Patients with hypomorphic *GINS3* variants present with MGS-like phenotypes. (**A**) Proband from Family 1 at 4 and 6 years of age. Note the frontal bossing, full lips, and posteriorly rotated ears. Lateral x-ray of the knee shows patella hypoplasia at age 4. (**B**) Patients from Family 2 at 16 year of age (P3) and at 24 years of age (P4), showing frontal bossing, full lips, prominent nose with long nasal root, and small ears posteriorly rotated ears. (**C**) Patient P5 at 4 years of age showing frontal bossing, full lips, micrognathia, small ears, and clinodactyly. (**D**) Patient P7 at 10 years of age showing frontal bossing, full lips, and prominent nose with long nasal root.

Seven individuals (patient 1 [P1] to P7) with MGS-like phenotypes of unknown genetic etiology were identified in 5 unrelated families (Figure 1A; patient characteristics are detailed in Supplemental Table 1; supplemental material available online with this article; https://doi.org/10.1172/jci.insight.155648DS1). The proband in the first family presented with prenatal and postnatal growth restriction, mild facial dysmorphisms, and frequent upper respiratory tract infections (Figure 1B, Figure 2A, and Supplemental Table 1; measurements 2 SD below or above average are considered abnormal; see Methods for calculation of growth parameters). By 4 years of age, he was developmentally appropriate, short (height –5.2 SD) with a head circumference at –2.2 SD and affected by chronic nonbloody diarrhea. Cytogenetic analyses on peripheral blood from the patient indicated overall genomic stability, with karyotype, sister chromatid exchange rates, immunological profile, and telomere length considered as normal (Supplemental Figure 1, A–E). The mother of P1 is 175 cm in height, while his father is 182 cm in height. The midparental height (predicted adult height of P1) is 185 cm, indicating that the short stature of P1 is not a result of reduced parental height.

P3 was initially investigated for MGS in view of short stature, multiple café au lait macules, small patella, and microtia (Figure 1B, Figure 2B, and Supplemental Table 1). He suffers from chronic respiratory infections and moderate neutropenia. At 16 years of age, he had mild cognitive delay, was short (height –2.1 SD), and was microcephalic (occipito-frontal head circumference [OFC] –3.4 SD). His 2 siblings, P2 and P4, were subsequently identified with similar physical characteristics (Figure 1B, Figure 2B, and Supplemental Table 1). P2 is short (height –2.2 SD), microcephalic (OFC –3.6 SD), and has a mild cognitive disability. P4 is also short (height –2.1 SD) and has a small OFC measuring –2.4 SD.

P5 presented with prenatal and postnatal growth restriction (Figure 1B and Supplemental Table 1). He was born at full-term with subtle facial dysmorphia and developed recurrent pneumonitis as a neonate that persisted into infancy without an identified etiology. His motor and cognitive skills developed appropriately; however, he suffered several asthma crises and was subsequently found to be neutropenic at 3 months of age. He had bilateral glaucoma, laryngomalacia, atrial septal defect (ASD) secundum, bilateral inguinal hernia, and undescended testes, which required orchidopexy. A brain CT scan showed bilateral subcortical and basal ganglia calcifications. On his latest assessment at 4 years of age, he was short (height –2.4 SD) and microcephalic (OFC –4.2 SD). He had frontal bossing, full lips, micrognathia, small mouth, prominent occiput, abnormal posteriorly rotated small ears, and small digits with clinodactyly (Figure 2C). He also had persistent neutropenia and lymphocyte markers indicating B lymphopenia, but his hemoglobin and platelet levels were normal. Neurological and hearing examinations and abdominal ultrasound were unremarkable. His parents are second cousins with no family history of dwarfism or congenital malformation.

P6 attained motor and cognitive milestones normally, but her growth parameters have consistently remained below average (height and weight –1.1 SD at most recent examination; Figure 1B and Supplemental Table 1). She has neutropenia and recurrent mouth ulcers with and without febrile episodes. She also had chronic suppurative otitis media, and a skull CT scan showed bilateral chronic mastoiditis. Two separate BM analyses showed normal cellularity with no maturation arrest and no dysplasia. Her parents are first cousins with no family history of dwarfism, MGS, or congenital malformation.

P7 presented with prenatal and postnatal growth restriction (Figure 1B and Supplemental Table 1) and was kept on oxygen support for a week after birth. Upon follow-up, she was underweight due to poor feeding. She had recurrent bronchiolitis during her first year of life. She attained motor and cognitive milestones normally. At her last assessment at 10 years of age, she was short (height –4.5 SD) and microcephalic (OFC –6.4 SD). She showed facial dysmorphia, including a prominent nose, frontal bossing, long nasal root, full lips, and small ears (Figure 2D). White blood cells were normal; however, her absolute neutrophil count ranged from 600 to 900. Other blood indices were normal. Echocardiography, abdominal ultrasound, trace element analyses, vitamin assays, and growth hormone analysis were unremarkable. Her parents are distant cousins. The height of her mother is 144 cm and that of her father is 170 cm. The midparental height for P7 is 151 cm (–2 SD), which suggests that the short stature of P7 may be partially attributable to parental genetic factors. P7 has a 24-year-old sister who has similar facial characteristics, chronic neutropenia, and severe growth retardation, but she has normal cognitive functions.

Exome sequencing was performed to investigate the underlying genetic etiology of the MGS-like symptoms of P1. Given the lack of family history, the disease was predicted to be autosomal recessive or de novo. While no genes had coding de novo or rare homozygous coding variants, 6 genes had candidate compound heterozygous variants (using a < 0.1% rarity cutoff; see Methods). For 5 of these, at least 1 variant was relatively common and had been seen to be homozygous in at least 1 presumed healthy individual in gnomAD (35). The remaining candidate was *GINS3*; variants were *GINS3* (NM_022770) c.71A>G, p.(Asp24Gly) (NC_000016.10:g.58392672A>G), and c.245G>A, p.(Arg82Gln) (NC_000016.10:g.58403159G>A). For the siblings of Family 2 (P2–P4), HomozygosityMapper (36) determined the candidate autozygome that is shared exclusively between the 3 affected siblings. Exome sequencing identified *GINS3* c.70G>A, p.(Asp24Asn) (NC_000016.10:g.58392671G>A), within the autozygome. Clinical exome sequencing was initially performed on families 3–5 (P5–P7) and were reported as negative. Revisiting these data allowed us to identify homozygous variant in *GINS3* c.71A>G, p.(Asp24Gly) (NC_000016.10:g.58392672A>G). Karyotype and chromosomal array were unremarkable in these patients.

Sanger sequencing further demonstrated the compound heterozygous *GINS3* D24G/R82Q variant in P1 and the carrier status of both parents, as well as the homozygosity in P3 and heterozygosity in the mother (Supplemental Figure 1F). The D24G/N variants have not previously been observed (for a list of databases searched,see Methods) and are predicted to be deleterious based on in silico analyses performed using SIFT (37), PolyPhen-2 (38), and CADD (39) (Supplemental Figure 1G). The R82Q variant has been observed in only 6 of 251,482 alleles in gnomAD v2.1.1; PolyPhen-2 and CADD predictions suggest potential deleterious impact, while the SIFT prediction suggests that this variant is tolerated.

**Figure 3:**
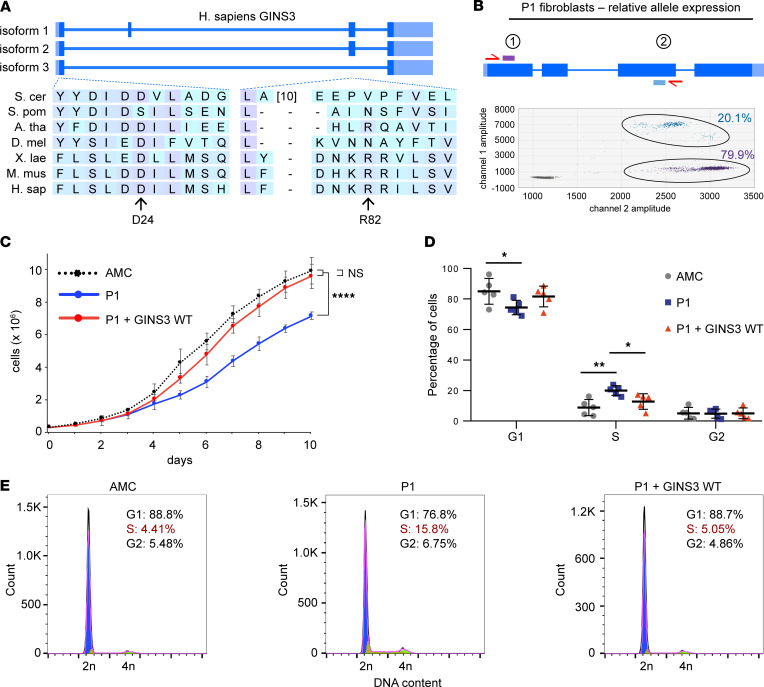
Patient-derived fibroblasts expressing GINS3 variants present cell cycle and proliferation defects. (**A**) *GINS3* variants affect conserved regions of the encoded protein. Representation of the 3 isoforms encoded by the *GINS3* gene and multispecies sequence alignment of regions containing pathogenic *GINS3* variants are shown. (**B**) Digital droplet PCR (ddPCR) analysis comparing the relative expression of the c.71A>G (p.D24G) and c.245G>A (p.R82Q) alleles in P1 primary fibroblasts. The position of the TaqMan probes is shown in purple and light blue. (**C**) Proliferation of P1 fibroblasts ± lentiviral delivery of WT *GINS3* compared with age-matched control (AMC) fibroblasts. Data were collected from 3 independent experiments and analyzed by 2-way ANOVA. (**D**) Cell cycle distribution of P1 fibroblasts ± lentiviral delivery of WT *GINS3* and AMC cells. Values were obtained by flow cytometry analysis of DNA content from 5 independent experiments and analyzed using a 2-way ANOVA. (**E**) One representative flow cytometry experiment is presented. For details on experimental procedures, see Methods. *P* values were adjusted for multiple comparisons where appropriate (see Methods). *P < 0.05, **P < 0.01, ****P < 0.0001.

*GINS3* encodes 3 main alternatively spliced isoforms (Figure 3A, top): all isoforms contain exon 1 (which encodes aspartic acid 24 ([D24]), but exon 3 (which encodes R82) is absent in isoform 3. This meant that there could be unequal representation of the D24G and R82Q GINS3 variants in P1 compound heterozygote cells. In line with GTEx (40) expression data from various tissues (ENSG00000181938.13, dbGaP accession phs000424.v8.p2), our reverse transcription PCR (RT-PCR) showed predominant expression of isoforms 2 and 3 in P1, P1 parental, and age-matched control (AMC) primary fibroblasts (Supplemental Figure 2A). Digital droplet PCR–based (ddPCR-based) allele expression analysis, using a pair of TaqMan probes each recognizing 1 of the 2 mutant *GINS3* alleles, also showed that the allele encoding D24G predominates over the allele encoding R82Q in P1 fibroblasts (Figure 3B). Expression analyses using additional probes in ddPCR experiments (Supplemental Figure 2B) demonstrated that, for P1, the D24G allele is represented in half of the *GINS3* transcripts and that less than a fifth of the *GINS3* transcripts harbor the R82Q variant (Figure 3B and Supplemental Figure 2B). Lower R82Q expression is due to predominant expression of isoform 3 that lacks the exon encoding R82Q. Analysis of PCR-amplified *GINS3* mRNA using mass spectrometry–based (MS-based) quantification (Supplemental Figure 2C) confirmed the predominant expression of transcripts encoding the D24G substitution.

### GINS3 variants compromise proliferation and cell cycle progression.

Primary fibroblasts were isolated from skin biopsies of P1 and cultured ex vivo. P1- and AMC-derived fibroblasts had a mean diameter of 16.2 ± 5 μm and 16.3 ± 5 μm, respectively, suggesting that the patient’s failure to grow is not due to reduced cell size (Supplemental Figure 3A). Interestingly, P1 cells presented significantly reduced proliferation in comparison with AMC fibroblasts (Figure 3C), and this process was reversed upon lentivirus-based expression of WT GINS3 (Figure 3C and Supplemental Figure 3, B and C). In contrast, expression of WT GINS3 had no effect on the growth of AMC fibroblasts (Supplemental Figure 3, B and C). Flow cytometry analysis of DNA content indicated a significantly larger proportion of primary P1 fibroblasts in S phase in comparison with AMCs (average of 8.84% S phase in AMC versus 19.96% in P1 fibroblasts; Figure 3, D and E), suggesting delayed progression/completion of DNA replication in P1 cells. Importantly, we observed significantly reduced accumulation of P1 fibroblasts in S phase upon lentivirus-based expression of WT GINS3 (average of 12.77% S phase in infected P1). The above data suggest that *GINS3* variants present compromised function, leading to defective proliferation and S phase progression in P1 fibroblasts.

### GINS3 variants impact gene transcription but not chondrocyte differentiation.

*GINS3* has no known role in gene expression; therefore, variants of this gene are not expected to directly impact gene transcription. We nevertheless performed an RNA-Seq experiment (Supplemental Figure 4A and Supplemental Data 1) to formally evaluate whether P1 cells might present abnormal expression of genes influencing cell or organismal growth. Some transcriptional changes were observed between primary fibroblasts isolated from P1 (D24G/R82Q) and the unaffected parents (WT/D24G and R82Q/WT). Of the 220 genes that had ≥ 4-fold difference and adjusted *P* ≤ 0.05, 83 were related to the “anatomical structure morphogenesis” gene ontology term (Supplemental Figure 4B). An enrichment in genes related to collagen-containing extracellular matrix (e.g., *COL23A1*, *TGM2*, *LAMC2*, *COL4A4*, *PRG4*) was also observed. These genes are normally expressed in chondrocytes and were downregulated in P1 versus AMC. Chondrocytes are the only cell type present in cartilage and are needed for the development of the skeletal system through endochondral ossification (41).

We sought to evaluate if the above-described changes in gene expression might cause defects in chondrocyte differentiation. P1 and AMC fibroblasts were differentiated into chondrocytes, and histological analyses were performed to evaluate formation of collagen fibers, as well as the accumulation of proteoglycans and mucins (Supplemental Figure 4C). We found that extracellular matrix molecules were secreted at similar levels by chondrocytes generated from control or P1 fibroblasts, and that expression of early (*SOX9*, *PTHLH*) and late (*COL2A*, *COL10A*) genes associated with chondrogenic differentiation was not significantly different in P1 versus AMC cells (Supplemental Figure 4D). Therefore, while the expression of certain genes needed for cartilage development was reduced in P1 fibroblasts in comparison with those originating from unaffected parents, chondrogenic differentiation itself was not compromised in P1 cells in vitro. Overall, the above findings suggest that the phenotypes observed in P1 are unlikely to be caused by abnormal gene expression leading to aberrant chondrogenic differentiation.

### D24N/G substitutions affect protein half-life and the GINS subcomplex.

**Figure 4:**
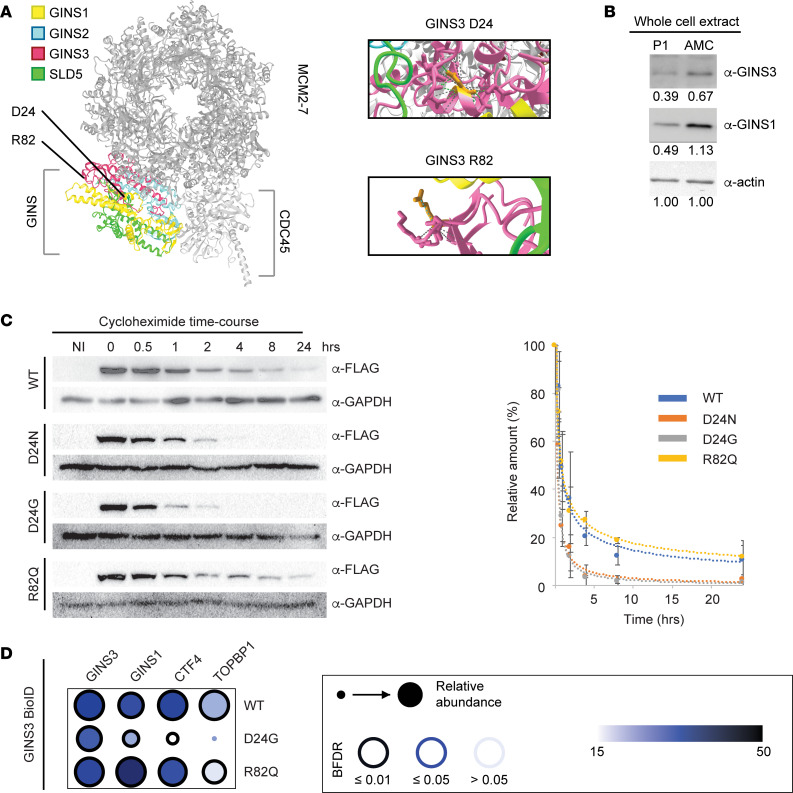
GINS3 D24 substitutions affect the protein half-life and protein-protein interactions at the replisome. (**A**) Position of the affected GINS3 residues within the cryoEM structure of the human CMG DNA helicase (PDB ID:6XTX; ref. 17). An enlarged view of the 2 residues is shown on the left. (**B**) Immunoblotting reveals decreased GINS3 and GINS1 levels in P1 primary fibroblasts in comparison with age-matched control (AMC) cells. (**C**) Half-life analysis of WT and GINS3 variant proteins. Exogenous FLAG-tagged GINS3 expression was induced in isogenic HEK293 Flp-In T-REx cells after which protein synthesis was halted by addition of cycloheximide in the culture media. The plot on the left is a representative example of 3 experimental replicates. NI, not induced. (**D**) Summary of results obtained in BioID analyses (see main text and Methods). For details on experimental procedures, see Methods.

Substitutions at D24 of the GINS3 protein are predicted to be detrimental to the stability of the GINS subcomplex, since this residue is buried within the complex and interacts with surrounding residues (Supplemental Figure 1G and Figure 4A). We found that protein levels for both GINS3 and GINS1 were lower in P1 fibroblasts as compared with AMC cells, consistent with destabilization of the complex (Figure 4B). We next generated isogenic HEK293 Flp-In T-REx cells (42) stably expressing, in a doxycyclin-inducible manner, the WT or variant proteins fused to a dual tag containing a Flag epitope and BirA* biotin ligase (43) (see Methods). While both WT and variant proteins were exclusively nuclear (Supplemental Figure 5A), GINS3 D24G protein expression was lower than that of WT and R82Q GINS3 (Supplemental Figure 5B). Consistently, inhibition of protein synthesis by cycloheximide treatment revealed that the D24N/G GINS3 proteins had a shorter half-life (~24 minutes) than that of the WT and R82Q proteins (~48 minutes; Figure 4C). We further found that GINS1 and GINS4 could be coimmunopurified with WT and R82Q GINS3, but not with the D24G variant (Supplemental Figure 5B). Together, these results indicate that GINS3 D24G/N variants present reduced stability and are not incorporated efficiently into the GINS complex.

To further validate the effect of MGS-associated variants on the interactome of GINS3, we performed proximity-based labeling of GINS3-associated proteins using BioID (43, 44). Cells expressing GINS3 proteins fused to the BirA* biotin ligase were incubated in the presence of excess biotin; biotinylated proteins were then isolated from cells and identified by mass spectrometry (Figure 4D and Supplemental Data 2). The R82Q variant and WT proteins presented similar interaction profiles. In contrast, the D24G variant presented drastically reduced associations with the replisome components TOPBP1, CTF4, and GINS1. Overall, the above data indicate that the GINS3 D24G variant affects the integrity and protein composition of the replisome progression complex.

### Cells expressing variant GINS3 present altered DNA replication.

**Figure 5:**
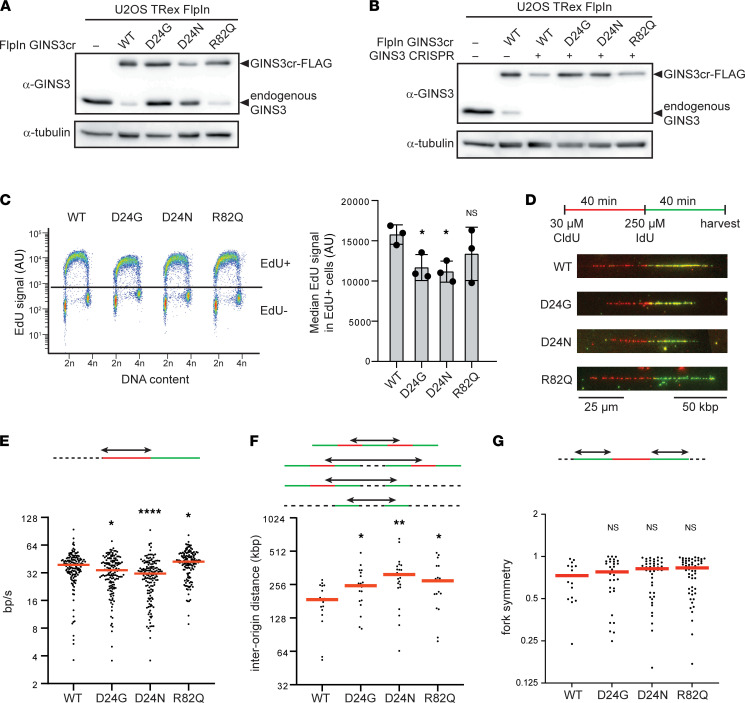
DNA replication dynamics are altered in U2OS T-REx Flp-In *GINS3*-KO cells expressing MGS-associated variants. (**A** and **B**) Western blots showing levels of GINS3cr-FLAG construct and endogenous GINS3 expressed before (**A**) and after (**B**) disruption of the endogenous *GINS3* gene by CRISPR-Cas9. For each cell line, a minimum of 3 independent protein extractions and 3 independent Western blots were performed; a representative example is shown. (**C**) Sample flow cytometry plots showing gating strategy for EdU^+^ cells (left) and bar graph of median EdU signal intensity in EdU^+^ cells (right). Three cell lines of each genotype were pulsed with EdU for 30 minutes and analyzed. Bars represent mean ± SD, and statistical analyses were performed using 2-tailed *t* tests. (**D**) Schematic summary of thymidine analog treatments for the DNA fiber experiment with sample fibers shown below. (**E**–**G**) Scatter plots showing median fork speed (**E**), interorigin distance (**F**), and fork symmetry (**G**). Red bars indicate median, and statistical analyses were performed using 2-tailed *t* tests. The experiment was performed twice; 1 representative replicate is shown. For details on experimental procedures, see Methods. *P* values were adjusted for multiple comparisons where appropriate (see Methods). *P < 0.05, **P < 0.01, ****P < 0.0001.

To study the effects of variant GINS3 on DNA replication in an isogenic model system, CRISPR/Cas9-resistant FLAG-tagged versions of WT or variant *GINS3* (expressing GINS3cr-FLAG) were introduced in U2OS cells using the Flp-In T-REx system (see Methods). Interestingly, expression of WT or R82Q GINS3cr-FLAG, but not D24G or D24N GINS3cr-FLAG, resulted in reduced levels of endogenous GINS3 (Figure 5A). Based on previous results (Figure 4D and Supplemental Figure 5B), we speculate that incorporation of WT and R82Q GINS3cr-FLAG proteins, but not the D24G/N variants, in the GINS complex might displace a fraction of endogenous proteins from the complex, thereby destabilizing them.

The endogenous copy of *GINS3* was disrupted in isogenic GINS3cr-FLAG–expressing cell lines using CRISPR-Cas9, rendering these GINS3-KO cells reliant on ectopically expressed GINS3 for survival (GINS3 KO causes cell lethality; ref. 45). Three GINS3-KO cell lines were isolated for each GINS3 variant. GINS3-KO cell lines expressing D24G or D24N GINS3 showed higher steady-state levels of GINS3 than those expressing WT or R82Q GINS3 (Figure 5B), suggesting that cells might adapt to functional defects in D24G/N GINS3 through selection for cells that express these variants at a higher level. Cell doubling times were assessed for 3 independent clones of each genotype, and no significant difference was observed between cell lines (Supplemental Figure 6); this is in contrast to results obtained with patient cells (Figure 3C), in which doubling time in patient cells was reduced, and this further suggests that some level of adaptation to the GINS3 variants occurred in the model cell lines.

To determine whether the GINS3 variants affected DNA replication, GINS3-KO cells complemented via ectopic expression of either WT or variant GINS3 were pulsed with the nucleoside analog EdU and analyzed by flow cytometry. No obvious differences in the fraction of cells in G1, S, or G2/M phases of the cell cycle were observed between cell lines (Supplemental Figure 7 and Supplemental Figure 8, top). However, cell lines expressing D24G or D24N GINS3 showed a significantly decreased rate of EdU incorporation per cell compared with those expressing WT or R82Q GINS3 (Figure 5C), suggesting that D24G and D24N GINS3 variants impair DNA replication.

Global DNA replication/S phase progression reflects both the speed at which replication forks progress and the number of active origins of replication. These 2 parameters were investigated using DNA fiber analyses. GINS3-KO cells were pulsed with CldU for 40 minutes followed by IdU for 40 minutes. DNA from cells was combed onto silanized slides and imaged by immunofluorescence microscopy, and CldU/IdU track length was measured (Figure 5D). Replication fork speed in cell lines expressing D24G or D24N GINS3 (33.9 bp/s and 30.8 bp/s, respectively) was significantly reduced as compared with those for cell lines expressing GINS3 WT (38.1 bp/s), while fork speed measurements for cell lines expressing GINS3 R82Q (41.6 bp/s) were greater than those of cell lines expressing GINS3 WT (Figure 5E). Interorigin distance (IOD) was found to be increased in cells expressing GINS3 variants compared with WT (262 kbp, 315 kbp, and 277 kbp for GINS3 D24G, D24N, and R82Q, respectively, compared with 195 kbp for GINS3 WT; Figure 5F). Overall, these data indicate that GINS3 variants alter DNA replication fork progression and impair the establishment or activation of origins.

DNA replication progression defects may be a cause or a consequence of elevated endogenous DNA damage, leading to replicative stress. Such endogenous DNA lesions can cause replication fork stalling and lead to asymmetrical advancement of a fork on one side of the origin of replication relative to the other, especially if fork restart/repair mechanisms are impaired. Fork symmetry was found to be similar in GINS3-KO cell lines expressing WT or variant GINS3, suggesting that GINS3 variants do not cause significant elevation in the abundance of endogenous DNA lesions relative to WT and do not significantly impair the restart of forks after stalling (Figure 5G). Levels of chromatin-bound Replication Protein A complex (RPA) and of phosphorylated histone H2AX (γ-H2AX), which are both well-known markers of DNA replication stress and associated DNA damage (46), were also assessed using a flow cytometry–based assay (Supplemental Figures 7 and 8). No significant differences were observed in the accumulation of RPA or γ-H2AX on chromatin, either in the presence or absence of exogenous replicative stress induced by hydroxyurea, suggesting that expression of GINS3 variants does not impair the ability of the cell to mitigate the deleterious consequences of DNA replication stress.

### GINS3 variant expression cause growth and cell cycle progression defects in yeast.

**Figure 6:**
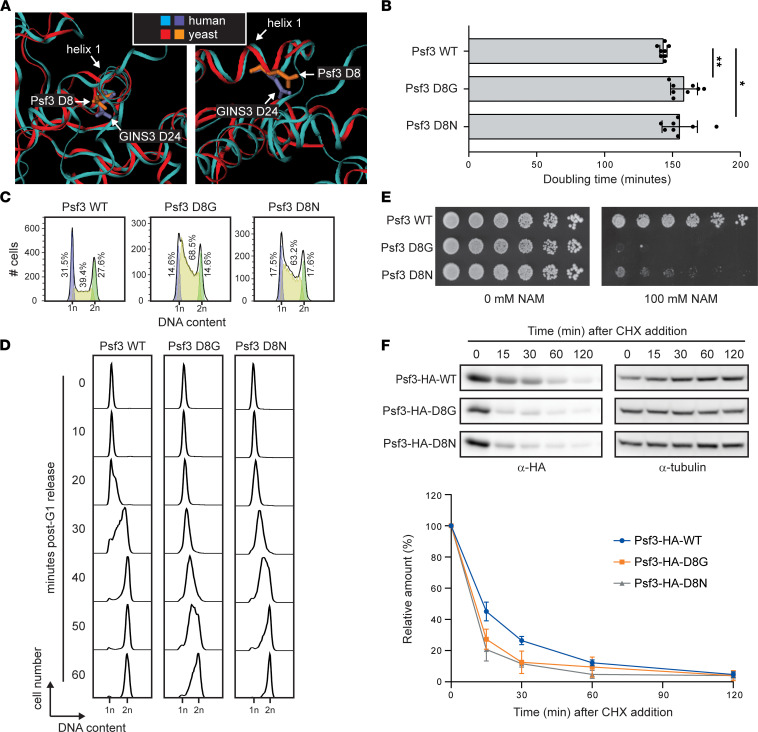
Yeast models expressing GINS3 D24G or D24N variants present defects in cell growth and protein stability. (**A**) Structural alignments of human GINS3 (PDB entry 2Q9Q) and yeast Psf3 (PDB entry 5U8S) proteins. A view down helix 1 (left) and a side view of helix 1 (right) are shown. Human GINS3 is in cyan, with residue D24 in ice blue; yeast Psf3 is in red, with residue D8 in orange. (**B**) Cell population doubling time, as measured by monitoring OD_630_ of 8 cultures of each genotype during exponential growth. Results are representative of 3 independent experiments, and statistical analyses were performed using 2-tailed *t* tests. (**C**) Cell cycle profiles of asynchronously growing (AS) cultures were assessed by flow cytometry. The experiment was performed twice; a representative replicate is shown. (**D**) Cells were synchronized in G1 by α factor arrest and were then released into S phase. Samples were fixed every 10 minutes after release and cell cycle progression assessed by flow cytometry. The experiment was performed in duplicate; a representative replicate is shown. (**E**) Serial 5-fold dilutions of yeast were grown on solid media in the presence or absence of nicotinamide (NAM) at 30°C for 72 hours. The experiment was performed in duplicate; a representative replicate is shown. (**F**) Exponentially growing yeast cultures were treated with 100 μg/mL cycloheximide (CHX). Samples were removed for protein extraction at the indicated times after CHX addition. Psf3 protein levels were assessed by immunoblotting. A representative blot is shown, and the results from 3 biological replicates are summarized in the graph. For details on experimental procedures, see Methods. *P* values were adjusted for multiple comparisons where appropriate (see Methods). *P < 0.05, **P < 0.01

DNA replication mechanisms are evolutionarily conserved across eukaryotes, with most proteins involved having direct homologs in species ranging from yeast to human; the yeast homolog of GINS3 is called Psf3. The region of GINS3 containing D24 is highly conserved, with yeast Psf3 D8 being the equivalent of human GINS3 D24, both in sequence and structural alignments (Figure 3A and Figure 6A). In contrast, the region of GINS3 containing residue R82 is not conserved in yeast (Figure 3A) and was, therefore, not further investigated in this organism.

Mutations were introduced in the endogenous *PSF3* gene to generate haploid yeast strains expressing D8G or D8N Psf3 (equivalent to human D24G and D24N GINS3, respectively. Analysis of doubling time in liquid culture revealed that cells expressing mutant Psf3 proliferate significantly more slowly than cells expressing Psf3 WT (Figure 6B). DNA content analysis by flow cytometry also indicated that cells expressing mutant Psf3 accumulate in S phase, with 68.5% of Psf3 D8G and 63.2% of Psf3 D8N cells in S phase, compared with 39.4% of Psf3 WT cells (Figure 6C). Consistently, upon release from α factor–mediated arrest in G1, cells expressing mutant Psf3 displayed slow progression through S phase compared with those expressing Psf3 WT (Figure 6D). Compared with cells expressing Psf3 WT, cells expressing mutant Psf3 grew poorly in the presence of nicotinamide, a compound that sensitizes yeast to DNA replication defects (47) (Figure 6E). Taken together, these results indicate that Psf3 D8G and Psf3 D8N are hypomorphic, leading to reduced proliferation, delayed progression through S phase, and increased sensitivity to DNA replication stress.

To determine whether, as is the case in human cells, compromised function of Psf3 D8G and Psf3 D8N is accompanied by decreased protein stability, we introduced plasmids encoding an epitope-tagged version of Psf3 (Psf3-3HA) in yeast strains in which the endogenous copy of *PSF3* was deleted. No obvious changes in steady-state Psf3 levels were observed between cells expressing WT and those expressing mutant Psf3 (Figure 6F). However, upon cycloheximide-induced inhibition of protein synthesis, Psf3 D8G and Psf3 D8N levels decreased more rapidly than those of Psf3 WT, suggesting that these Psf3 mutants are unstable compared with Psf3 WT (Figure 6F, bottom panel), as was observed for the GINS3 D24G/N variants in human cells.

### Mouse model of GINS3 D24N shows embryonic lethality and increased senescence.

**Figure 7:**
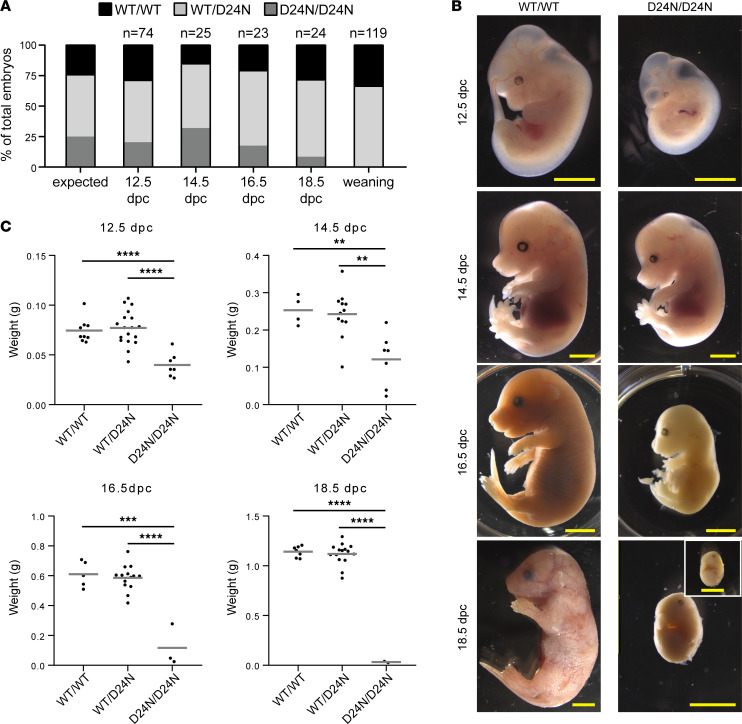
Homozygosity for *Gins3^D24N^* allele leads to intrauterine growth restriction and embryonic lethality in mice. (**A**) Genotype distribution of embryos and newborn mice resulting from *Gins3^WT^/Gins3^D24N^* × *Gins3^WT^/Gins3^D24N^* matings. (**B**) Images of *Gins3^WT^/Gins3^WT^* (WT/WT) and *Gins3^D24N^/Gins3^D24N^* (D24N/D24N) embryos at 12.5, 14.5, 16.5, and 18.5 dpc. Scale bar: 2 mm. (**C**) Weight (g) of embryos at 12.5, 14.5, 16.5, and 18.5 dpc. For details on experimental procedures, see Methods. *P* values were from 2-tailed *t* tests adjusted for multiple comparisons (see Methods). **P < 0.01, ***P < 0.001, ****P < 0.0001

We next evaluated the impact of *GINS3* variants on growth and development in a murine model. The GINS3 D24N variant was chosen for this model as P2, P3, and P4 were the first patients identified that were homozygous for a *GINS3* variant in our study, meaning that there was reason to believe that a homozygous mouse model might be viable. CRISPR-Cas9 was used to mutate the endogenous *Gins3* locus to generate heterozygous *Gins3^WT^*/*Gins3^D24N^* mice. These mice were viable and fertile. Pairs of *Gins3^WT^*/*Gins3^D24N^* mice were crossed with the goal of obtaining homozygous *Gins3^D24N^*/*Gins3^D24N^* mice; however, none of the 119 pups genotyped at weaning were found to be *Gins3^D24N^*/*Gins3^D24N^* (0 of 119; Figure 7A). Since no mortality was noted in the pups with careful mouse husbandry, these data suggest that the *Gins3^D24N^*/*Gins3^D24N^* genotype is either embryonic lethal or causes death at a very early postnatal stage. To characterize the developmental effects of the *Gins3^D24N^* variant, crosses were performed, and embryos were harvested at 12.5, 14.5, 16.5, and 18.5 dpc (Figure 7B). Genotype analysis showed that the percentage of *Gins3^D24N^*/*Gins3^D24N^* embryos is less than that expected based on Mendelian inheritance (25%) from 16.5 dpc onward. Examination of embryos revealed fetal resorption of *Gins3^D24N^*/*Gins3^D24N^* embryos at 14.5, 16.5, and 18.5 dpc, which is indicative of embryonic lethality. At all stages, the weight of *Gins3^D24N^*/*Gins3^D24N^* embryos was strongly reduced compared with WT and heterozygous ones (Figure 7C). Together, these results show that mouse embryos homozygous for the *Gins3^D24N^* variant have an intrauterine growth restriction (IURG) phenotype and die during embryonic development.

**Figure 8:**
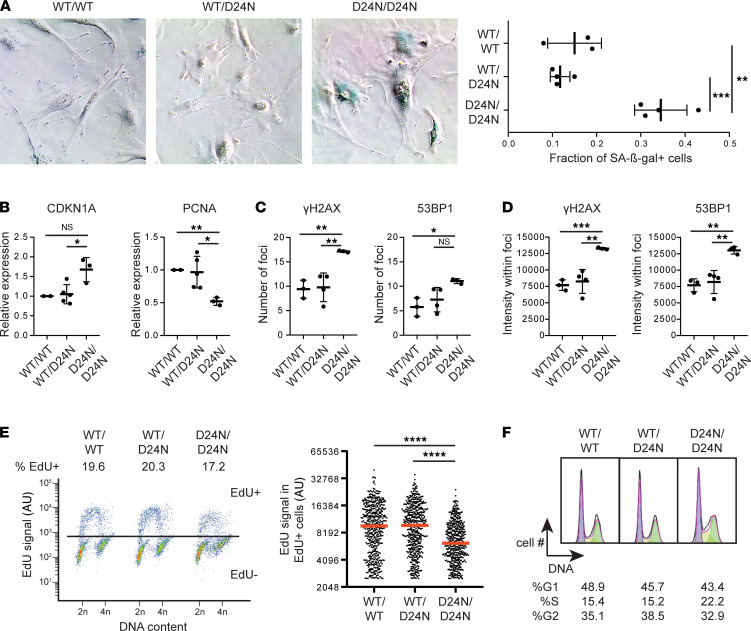
Mouse embryonic fibroblasts homozygous for *Gins3^D24N^* allele show increased senescence and decreased DNA replication. (**A**) Senescence-associated β-galactosidase staining was assessed by bright-field microscopy in 3 separate experiments. Each point in the graph represents a separate MEF cell line. Results were combined by taking the mean of cell lines assayed in multiple experiments. A minimum of 500 cells were analyzed per experiment, and statistical analyses were performed using 2-tailed *t* tests. Original magnification, 100×. (**B**) Expression of senescence-associated genes, as measured by qPCR, normalized to *Gins3^WT^/Gins3^WT^* cell line. Each point represents an independently isolated cell line. The experiment was performed twice independently, and statistical analyses were performed using 2-tailed *t* tests. (**C** and **D**) Cells were fixed and immunostained with antibodies against the indicated protein. Foci number (**C**) and intensity (**D**) were assessed for a minimum of 50 cells from each cell line as described in Methods, and statistical analyses were performed using 2-tailed *t* tests. (**E**) Asynchronous cultures of primary MEFs were pulsed with 10 μM EdU for 2 hours prior to fixation. EdU content was assessed by flow cytometry (left). EdU levels in EdU^+^ cells are shown in scatter plot (right); bar represents median, and statistical analyses were performed using 2-tailed *t* tests. Experiment was performed in duplicate; a representative replicate is shown. (**F**) Cell cycle profile of cells from EdU experiment shown in **E**. *P* values were adjusted for multiple comparisons where appropriate (see Methods). For details on experimental procedures, see Methods. *P < 0.05, **P < 0.01, ***P < 0.001, ****P < 0.0001.

To examine how *Gins3^D24N^* homozygosity affects cell growth and DNA replication, mouse embryonic fibroblasts (MEFs) were isolated at 12.5 dpc. Cell culture observations indicated *Gins3^D24N^*/*Gins3^D24N^* cells ceased to proliferate much earlier than *Gins3^WT^*/*Gins3^WT^* or *Gins3^WT^*/*Gins3^D24N^* cell lines (i.e., after 3 or 4 passages compared with 9 or 10 passages, respectively). Senescence-associated β-galactosidase staining after 3 passages revealed significantly increased staining in *Gins3^D24N^*/*Gins3^D24N^* cell lines, indicating that these cells probably proliferate poorly due to premature senescence (Figure 8A). We also observed increased expression of CDKN1A, the mRNA encoding p21, and decreased expression of PCNA mRNA in *Gins3^D24N^*/*Gins3^D24N^* cell lines compared with WT or heterozygous cell lines, consistent with senescence induction (48) (Figure 8B). An increase in the number and intensity of foci of the DNA damage response markers γ-H2AX and 53BP1, which are well-known markers of cellular senescence (48), was also observed for *Gins3^D24N^*/*Gins3^D24N^* cell lines (Figure 8, C and D, and Supplemental Figure 9). Together, these results show that homozygous *Gins3^D24N^*/*Gins3^D24N^* MEFs are more prone to senescence than corresponding heterozygotes or WT cells.

Flow cytometry assays were performed to examine DNA replication and cell cycle dynamics in MEFs. Analysis of EdU incorporation showed that *Gins3^D24N^*/*Gins3^D24N^* cells that are in S phase and actively replicating their DNA (EdU^+^) incorporate less EdU than *Gins3^WT^*/*Gins3^WT^* or *Gins3^WT^*/*Gins3^D24N^* cell lines, consistent with decreased rate of DNA replication (Figure 8E). *Gins3^D24N^*/*Gins3^D24N^* cell lines also accumulated in S phase, likely due to compromised DNA replication (Figure 8F). Overall, these results show that *Gins3^D24N^*/*Gins3^D24N^* MEFs present DNA replication defects, which may contribute to their premature entry into senescence.

## Discussion

We have identified 7 patients with MGS from 5 unrelated families. These patients presented no variants in genes previously associated with MGS or any other form of primordial dwarfism; however, all patients present variants of the essential DNA replication gene *GINS3*, P1 being compound heterozygous (c.71A>G, p.[Asp24Gly], and c.245G>A, p.[Arg82Gln]) and P2 through P7 being homozygous (P2–P4 for c.70G>A, p.[Asp24Asn]; P5–P7 for c.71A>G, p.[Asp24Gly]). Our results using yeast, murine, and human models, as well as patient cells, clearly demonstrate that the identified *GINS3* variants are hypomorphic and lead to growth, cell cycle progression, and DNA replication defects. Overall, this experimental evidence leads us to propose that hypomorphic *GINS3* variants are a heretofore unreported cause of MGS.

The *GINS3* gene is essential for viability of human cells (45), and homozygous deletion of *Gins3* is embryonic lethal in mice (49). The GINS3 protein is a component of the CMG helicase, responsible for separating DNA strands to allow access for DNA polymerases during DNA replication. Several other components of the CMG helicase have previously been implicated in the etiology of MGS, including CDC45 (24, 25, 27), MCM5 (23), MCM3 (22), MCM7 (22), and, recently, GINS2 (28). Other components of the CMG helicase, including MCM4 and GINS1, have been linked to forms of primordial dwarfism similar to MGS, but they have been classified as clinically distinct due to the fact that they include immune deficiencies and/or adrenal insufficiency (31–34).

The molecular mechanisms explaining why some hypomorphic CMG variants lead to classic MGS, while others lead to forms of primordial dwarfism that include other phenotypes, are unclear. In the case of subunits of the GINS complex, GINS1 variants have been identified in patients with primordial dwarfism accompanied by immune deficiencies, specifically a NK cell deficiency and chronic neutropenia (33), while a biallelic GINS2 variant has been identified in a patient with typical MGS symptoms, namely primordial dwarfism, microcephaly, and small ears but no immunodeficiency (28). In some cases, specific variants within the same gene have been reported to lead to different clinical presentations, as observed for MCM7 (22). As previously reported for MGS patients (20, 50), phenotypic expression was found to be variable in our cohort of patients with *GINS3* variants. In particular, the height of P6 currently falls within a range that can be considered as normal for her age, while other clinical data — e.g., those regarding her ears and patellae, which would confirm MGS diagnostic — are unavailable. Indeed, we note that, since this and other patients from our cohort are from remote regions of the Middle East, regular clinical assessment and data collection was challenging. Nevertheless, clinical observations indicate that growth parameters of P6 have been consistently below average throughout her lifetime. Importantly, phenotypic variability is known to exist among MGS patients (50), with height ranging from –9.6 SD to –0.4 SD in certain cohorts (20). We also note that some patients of our cohort were seen to be immunodeficient, while others were not. As mentioned above, such phenotypic heterogeneity is typical of MGS patients (50), which suggests that CMG helicase-associated disorders are probably influenced by additional factors that contribute to the overall clinical presentation. More study of DNA replication dynamics during development is needed in order to better understand the mechanistic basis of these phenotypes. However, in the case of *GINS3* variants identified here, the embryonic lethality of homozygous mice complicates the study of such developmental defects.

While all patients carry at least 1 copy of variant *GINS3* expressing a protein with a substitution at residue D24, P1 also carries a *GINS3* allele expressing a protein in which glutamine is substituted for arginine 82 (R82Q). The R82 residue is not evolutionarily conserved in yeast and is situated in a random coil within the GINS3 protein that is not known to participate in any protein-protein interactions. The effects of the R82Q variant were apparently not as deleterious as those seen for the D24 variants, with protein stability being similar to that of WT and with interactions of the variant protein with other components of replication forks being similar to WT GINS3 in vivo. However, our DNA fiber assay data indicate that the R82Q variant causes a decrease in the number of active replication origins, which is accompanied by concomitant increase in replication fork speed. Reduced number of active replication origins has previously been shown to increase the availability of nucleotides, which typically promotes elevated replication fork speed (51). In the case of the R82Q variant, this inverse correlation between fork speed and origin number can be observed, although the precise mechanism through which the R82 residue might influence origin activity is currently uncharacterized. In the case of the D24 variants, both replication fork speed and origin number are decreased, suggesting that these parameters are directly affected by the D24 variants. The observed decrease in protein stability for the D24 variants might potentially explain, at least in part, the above-described effects on DNA replication dynamics, as GINS3 is essential for both origin firing and ongoing replication.

Despite the reduced severity of the molecular effects caused by the GINS3 R82Q variant, it does appear to contribute to the MGS phenotype of P1, as the mother of P1 is heterozygous for GINS3 D24G and does not show any features of MGS, consistent with an autosomal recessive mode of inheritance. Although the GINS3 R82Q variant did not negatively affect rates of DNA replication in our cell model, small perturbations in the function of GINS3 R82Q may manifest themselves at the organismal level only in the presence of other variants (e.g., D24G) and may have greater effects during development or within particular tissues. We also note that our human cell models used *GINS3* cDNA expressed under the control of a tetracycline-inducible promoter, meaning that any regulation of *GINS3* that depends on genomic context (i.e., transcriptional regulation, mRNA splicing) was not effectively modeled. Although no changes in protein levels between endogenously and exogenously expressed GINS3 were evident by immunoblotting, the possibility that slightly increased GINS3 expression from our tetracycline-inducible promoter could potentially mask the severity of the defects caused by R82Q cannot be excluded. Additionally, analysis of *GINS3* transcripts in P1, the parents of P1, and an AMC showed that the balance of *GINS3* isoforms was altered in cells expressing GINS3 R82Q. Indeed, while the overall abundance of *GINS3* transcripts in the cell is not altered, in cells encoding the GINS3 R82Q variant (i.e., the cells of P1 and the father of P1), expression of isoform 3, which excludes the exon encoding R82, is higher than that of isoforms 1, which include this exon. While it is unclear whether isoform 3 is fully functional and can compensate for isoform 1 and 2, it is possible that the above-described differential isoform expression might ultimately reduce any functional consequences of the R82Q variant.

The highly conserved nature of the CMG helicase allowed us to model the GINS3 D24 variants in other species. Modeling in yeast further confirmed the functional importance of the GINS3 D24 residue in DNA replication, with the phenotypes observed in yeast being fully consistent with those identified in human cells. In our mouse model, homozygosity for *Gins3^D24N^* was lethal either at an embryonic or early postnatal stage, which is intriguing since GINS3 D24N homozygosity is not lethal in humans. Homozygous deletion of *Gins3* in mice is reported to be embryonic lethal prior to 9.5 dpc (49), while we observed that embryos homozygous for *Gins3^D24N^* can survive to at least 18.5 dpc, supporting our hypothesis that the *Gins3^D24N^* variant is hypomorphic. We further showed that MEFs isolated from D24N homozygous embryos accumulated in S phase and showed decreased rates of DNA replication, consistent with our observations in the yeast and human cell systems. The D24N homozygous MEFs also showed an increased rate of senescence, which was not observed in human cells but is consistent with the lack of viability of this genotype in mice. Based on the above, it is tempting to speculate that DNA replication defects caused by GINS3 D24N contribute to elevated senescence in MEFs. It is unclear why developing mouse embryos are more sensitive to defects in CMG activity than human ones. Comparative studies of mouse and human genomes have identified a number of differences between the 2 organisms, including in DNA replication timing, gene expression, DNA regulatory elements, and epigenetic marks (52–54). Nevertheless, one possibility is that human cells harboring *GINS3* variants might be prone to senescence and reduce overall cell proliferation during development, leading to the failure to thrive that is often observed in MGS patients.

We note that, while gene variants that perturb DNA replication often lead to developmental problems, including forms of primordial dwarfism such as MGS, the precise molecular mechanism by which this occurs is unclear. Moreover, the reasons for the wide range of phenotypes associated with perturbation of DNA replication remain poorly understood. Further investigations aimed at linking developmental and DNA replication defects at the organismal level will be essential to better understand the molecular etiology of these rare syndromes.

## Methods

### Sequencing, rare variant identification, and variant validation.

Details regarding sequencing, rare variant identification and variant validation can be found in the Supplemental Methods. Human genome assembly GRCh38.p13 was used as the reference genome where positions in genomic DNA are indicated. Consanguinity was determined by personal communication and not by genetic means (i.e., homozygosity mapping).

### Clinical parameters.

Cytogenetic testing for chromosome breakage was done as previously described (55). For the sister chromatid exchange analysis, patient and control peripheral blood samples were cultured in MEM α/FBS for a total of 72 hours. Thirty-six hours prior to harvesting, 30 μg/mL BrdU (MilliporeSigma) was added to the cultures. Harvesting of cultures and slidemaking were performed according to standard cytogenetic techniques. Slides were aged by maintaining at room temperature for 5 days. Slides were then immersed in Hoechst 33342 Dye (final concentration 3 μg/mL) (Sigma) for 1.5 hours while protected from the light. After rinsing with PBS and coverslipping, slides were exposed to Black Light (365 nm) for 2 hours. Coverslips were removed, and slides were rinsed again with PBS and stained with Giemsa (MilliporeSigma). Sister chromatid exchanges were scored in 20 metaphase cells from the patient and control.

For the immune evaluations, the numbers of T, B, and NK lymphocytes were assessed at 7.5 years of age as previously done (56), and the diversity of CD4^+^ and CD8^+^ T cells was determined by flow cytometry analysis as previously described (57). An NK degranulation assay was performed by measuring surface expression of CD107A by flow cytometry, as previously reported (58). Telomere length was assessed by flow cytometric analysis of telomeric FISH (Flow FISH) using RepeatDx (59).

### Plasmids and site-directed mutagenesis.

For details of plasmid generation and site-directed mutagenesis, see Supplemental Methods.

### Cell culture and generation of cell lines.

Dermal primary fibroblasts were grown from skin-punch biopsies in DMEM medium (Corning) and then maintained in complete medium (DMEM supplemented with 10% FBS [Wisent], 2 mM L-glutamine, and 200 U/mL penicillin/200 μg/mL streptomycin). Generation and culturing of cell lines expressing variant forms of GINS3 is detailed in Supplemental Methods. Lentiviral particles were generated at the SickKids Proteomics, Analytics, Robotics & Chemical Biology Centre (SPARC). Lineages were regularly tested for mycoplasma.

### Cell size, growth curves, and cell cycle analyses.

To determine the cell diameter of the primary fibroblasts, cultured cells were trypsinized, and the cell suspension was analyzed using a Multisizer 4 Coulter Counter (Beckman-Coulter), as described (60). To obtain growth curves, primary fibroblasts at passage 15 were infected with lentiviral particles as described in the results and seeded the next day at 0.3 × 10^6^ cells per culture vessel. A culture vessel was harvested every day, over 10 days, and trypsinized cells were counted using a Countess II automated cell counter (Thermo Fisher Scientific). For primary cells, the cell cycle was determined by flow cytometry. To prepare samples, 2 × 10^6^ cells were washed with PBS, resuspended in 50 µL staining medium (1× HBSS [Thermo Fisher Scientific] containing 10 mM HEPES [pH 7.2], 2% calf serum [Wisent], 0.1% NaN3 [Millipore Sigma]) and then immediately fixed by adding 1 mL of 80% ice-cold ethanol while vortexing. Cells were recovered by centrifugation at 400*g* for 5 min at 4°C and resuspended in 500 μL HBSS supplemented with 2 mg/mL RNase A (Millipore Sigma). RNA was digested for 5 minutes at room temperature, after which 500 μL HBSS supplemented with 0.1 mg/mL propidium iodide and 0.6% tergitol (MilliporeSigma) was added. Samples were vortexed and incubated for 30 minutes at room temperature in the dark. Cells were centrifuged at 400*g* for 5 minutes at 4°C, and the cell pellet was resuspended in 500 μL staining medium with no serum. Samples were gently vortexed, filtered using a nitrex mesh, and data were collected on a LSR II flow cytometer (BD Biosciences) using CellFIT (Becton Dickinson).

### Allelic balance and gene isoforms.

Allelic balance was assessed at both the mRNA and protein level. For detailed methods, see Supplemental Methods.

### RNA isolation, quantitative PCR (qPCR), and RNA-Seq.

qPCR was performed with technical triplicates. RNA-Seq was performed from 3 separate patient fibroblast cultures and 1 replicate of fibroblasts derived from each of the 2 parents. For detailed RNA isolation, qPCR and RNA-Seq methods, see Supplemental Methods.

### Western blotting, immunoprecipitations, and immunolabeling.

Western blotting was done as previously described (61) using Hybond PVDF membranes (GE Healthcare) and the following antibodies: GINS3 (Abcam, AB177515), GINS1 (Abcam, AB183524), FLAG (MilliporeSigma, F1804), V5 (Bio-Rad, MCA1360GA), HA (12CA5; Abcam, AB1424), GAPDH (Santa Cruz Biotechnology Inc., sc-25778), and actin (Abcam, AB1801). Bands of interest were quantified by densitometry using ImageJ software (NIH).

For details of immunoprecipitation and immunolabeling procedures, see Supplemental Methods.

### BioID.

All BioID runs were performed in biological duplicates. For details of BioID procedure, see Supplemental Methods.

### Protein digestion and LC-MS/MS analysis.

For details of protein digestion and LC-MS/MS analysis, see Supplemental Methods.

### Cyclohexamide chase assay.

HEK293T Flp-In T-REx were seeded in 6-well plates at 40% cell confluency and induced with 1 μg/mL tetracycline for 24 hours to express exogenous GINS3-BirA* fusion proteins. After induction, cells were treated with 10 μg/mL cyclohexamide and harvested at the time points indicated in the figures. Cell pellets were flash frozen prior to protein extraction from experimental sets. For yeast cultures, cyclohexamide was added at 100 μg/mL to 0.4 OD yeast cultures incubated at 30°C with shaking. Samples were collected at indicated time points, and cell pellets were frozen at –20°C prior to protein extraction. Proteins were extracted using standard glass bead-trichloroacetic acid methods.

### Yeast strains, strain construction, and growth conditions.

Yeast strains used in this study were generated from the BY4743 strain background (MAT a/alpha; his3Δ1/his3Δ1; leu2Δ0/leu2Δ0; met15Δ0/MET15; LYS2/lys2Δ0; ura3Δ0/ura3Δ0) and cultured at 30°C in YPD medium (1% yeast extract, 2% peptone, 2% dextrose; all purchased from Bioshop Canada inc.) unless otherwise indicated. For details of yeast strain construction, see Supplemental Methods.

### Yeast doubling time and S phase progression.

Yeast experiments were performed essentially as previously described (47). For detailed procedures, see Supplemental Methods.

### Flow cytometry analysis of EdU incorporation.

Cells were pulsed with 10 μM EdU for 30 minutes; then, EdU incorporation and chromatin-bound RPA and γH2AX were quantified by flow cytometry as previously described (62). Antibodies used were anti-RPA70 (Abcam, ab79398, 1:200), anti-γH2AX (MilliporeSigma, 05-636, 1:200), Alexa Fluor 488 goat anti–mouse IgG (H+L) (Invitrogen, A11029, 1:200), and Alexa Fluor 594 goat anti–rabbit IgG (H+L) (Invitrogen, A11012, 1:200); click reaction was performed using Alexa Fluor 647 (Invitrogen, A10277, 1:200). Flow cytometry was performed using a BD LSRFortessa cell analyzer with BD FACSDiva software (BD Biosciences), and analysis was performed with FlowJo software (BD Biosciences, version 10) with the Watson (Pragmatic) model for cell cycle analysis.

### DNA combing analysis.

DNA combing was performed essentially as previously described (63, 64). For detailed procedure, see Supplemental Methods.

### Generation of Gins3^WT^/Gins3^D24N^ mice.

The human mutation NM_022770.2: c.70G>A, p.(Asp24Asn) was introduced into the mouse genome using the CRISPR/Cas9 method. The mice were generated by the McGill Integrated Core for Animal Modeling (MICAM; McGill University, Montreal, Quebec, Canada). Briefly, the sgRNA (Synthego), Cas9 protein (IDT, catalog 1081058), and ssODN (ultramer, IDT) were microinjected into the pronucleus of C57BL/6N mouse zygotes with concentrations of 50:50:30 ng/μL respectively. Embryos were subsequently implanted in CD-1 pseudopregnant surrogate mothers according to standard procedures approved by the McGill University Animal Care Committee (UACC). Oligonucleotides used were mGins3-gRNA and ssODN. After weaning, the mice were transferred to the Centre de Recherche du Centre Hospitalier Universitaire (CR-CHU) of Sainte-Justine Hospital. Mouse husbandry and experiments were done according to the approved animal user 732-NAGANO protocol no. 2021-3228 by the Coordonnatrice du Comité Institutionnel des Bonnes Pratiques Animales en Recherche (CIBPAR). This committee is following the guidelines of the Conseil Canadien de la Protection des Animaux (CCPA). The presence of the mutation was confirmed by Sanger sequencing using mGins3-D24Nseq-F and mGins3-D24Nseq-R.

### Senescence-associated β-galactosidase (SA-β-gal) assay.

For details of SA-β-gal assay, see Supplemental Methods.

### Statistics.

SDs of patients from average measurements were calculated at birth using the Fenton Growth Chart (65) and after birth using CDC Growth Charts (66). IUGR and SGA were defined as less than 10% of predicted weight for gestational age (67, 68). A comparison of proliferation rates in mammalian cells was done using 2-way ANOVA analysis, while changes in cell cycle were analyzed using a 2-tailed paired *t* test. For RNA-Seq, differential gene expression analysis was performed using DESeq2 (69). In RT-PCR experiments, 2-tailed unpaired *t* tests were performed on control and patient ΔCt values using GraphPad Prism version 8. For MS analyses, protein-protein association probabilities were determined by comparing GINS3 BioID results to that of the GFP and NLS negative controls using Significance Analysis of INTeractome (SAINT; ref. 70). Enrichment ratios were considered significant if they had a Bayesian FDR (BFDR) ≤ 5%. Changes in EdU incorporation, mouse embryo weight, SA-β-gal staining, MEF transcript levels and immunofluorescence were analyzed using 2-tailed unpaired *t* tests performed with GraphPad Prism version 8. Changes in replication fork dynamics were analyzed using 2-tailed unpaired *t* test performed with GraphPad Prism version 8. Throughout this work, when multiple comparisons were made using *t* test, significance was assessed using the Holm-Sidak method with α = 0.05; multiplicity adjusted *P* values obtained using this procedure are presented in relevant figures. *P* < 0.05 was considered significant.

### Study approval.

Written informed consent for the work done in this study was obtained from the families. Further approval was obtained by the Hospital for Sick Children Research Ethics Board (REB no. 1000057001) and Maisonneuve-Rosemont Hospital Research Center (project no. 2018-1057).

## Author contributions

PK, PMC, EIC, and HW conceived of and designed the study. RS, KDK, LD, JS, MS, ZNA, MDW, EG, KMB, SM, EAF, FA, ZUK, and FSA performed clinical analyses. KA, ST, PG, and ESD performed experiments with patient cells and HEK293 cells. SA and PGM performed experiments related to chondrocyte differentiation. KEY and MDW performed RNAseq experiments. ST, PG, and FB performed and analyzed BioID experiments. MEM performed experiments with U2OS cells and yeast. JR performed mouse experiments. RT and MEM performed experiments with MEFs. PK, PMC, EIC, HW, MEM, SM, and JR participated in the writing of the initial draft of the manuscript. All authors reviewed the manuscript.

## Supplementary Material

Supplemental data

Supplemental data set 1

Supplemental data set 2

## Version 1 05/23/2022

Electronic publication
